# 

**Published:** 1915-04-03

**Authors:** 


### CONTENTS.

PAGE

**The Medical Needs of the Army** ... ... 1

**The Easter Holiday** ... ... ... ... ... 2

**Hospital and Institutional News** ... ... ... 3

The War Office and the Poor Law—A Surgeon's Experiences as a Prisoner in Germany—Naval Surgeons for the Army—Claim of £15,000 against St. George's Hospital—Staff and War Salaries at Leicester—The New Chairman's Policy—Nerve Hospitals and Drugs—£150,000 for Two Hospitals—The New President of the Coroners' Society—The Special Medical Reports of the L.C.C. Education Department—Poor-Law Infirmaries and Medical Supplies—The London Mental Deficiency Committee—Poor-Law Formalities—Cardiff Mental Hospital and the War Office—The School Medical Service in Birmingham—The Ambulance Service for London—A Whole-Time Medical Service in Bermondsey—The Value of Health Visitors—"The Way of the Red Cross"—Mental Hospitals for the Wounded—Duty-Free Rectified Spirit in Hospitals—Refuse and the Health of London—Easter and the Drug Market—To Our Readers.

PAGE

**Tuberculin in Ophthalmic Practice** ... ... 9

The Method Adopted and Results.

**The Distinguished Service Order** ... ... ... 10

**A Medical Causerie** ... ... ... ... ... 11

**Round the World** ... ... ... ... ... 12

Fellow-Workers and the Roll of Honour.

**The "Notes" in the Annual Returns** ... ... 13

King Edward's Hospital Fund for London Statistics.

**Legal Notes for Institutional Workers** ... ... 14

Insurance Policies.

A New Field for Medical Witnesses.

**The Evolution of the Modern Hospital** ... ... 15

V.—Medical Education, Administration, and Finance (*continued*).

**The Editor's Letter-Box** ... ... ... ... 17

The Colony for Cripples, Chailey.

The Organisation of Hospital Fire Brigades.

[*See over.*]

### CONTENTS—(*continued*).

| | PAGE |
|---|---|
| **The Institutional Library** ... ... ... ... | 18 |
| Surgical Procedure. | |
| Mental Disorders. | |
| The S.P. Pocket Guide Series. | |
| **The Department of Modern Nursing** ... ... | 19 |
| XI.—The Royal Infirmary of Edinburgh. | |
| **From the Patient's Point of View** ... ... | 22 |
| A Territorial's "Internment" in an Isolation Hospital. | |
| **The Institutional Worker** ... ... (Suppl.) | 1 |
| III.—The Steward. | |
| Questions for April. | |
| Enquiries and Answers. | |
| The Sick and in Need. | |
| Miscellaneous. | |
| Acknowledgments. | |
| Answer to March Question: Noise in Institutions. | |

